# Assessment of Self-Esteem Among Tunisian Cannabis Users

**DOI:** 10.1192/j.eurpsy.2022.626

**Published:** 2022-09-01

**Authors:** S. Brahim, M. Henia, A. Haj Mohamed, M.H. Aoun, L. Zarrouk

**Affiliations:** 1 University Hospital of Mahdia, Psychiatry, chebba, Tunisia; 2 University Hospital of Mahdia, Tunisia., Psychiatry, mahdia, Tunisia

**Keywords:** self-esteem, Cannabis, Tunisia

## Abstract

**Introduction:**

Cannabis is among the most widely used substances in the world. it is associated with several mental health problems.

**Objectives:**

To assess self-esteem among a group of young Tunisian users of cannabis.

**Methods:**

The total study sample was composed of 137 participants, who took part of a transversal descriptive study during two months (January and February 2020).

**Results:**

In our study population, the cannabis consumers were young adults aged between 18 and 35 years old, with a male predominance of 71%. Among those users, 65.9% were single and 29.7% dropped out of school or experienced academic failure. On a socio-economic level, we concluded to a rate of 5.8% (lower class), 60.9% (middle class) and 33.3% (upper class). Besides, 40.8% were employed. In total, 23.2% had a psychiatric history. Furthermore, the use of other substances was also prominent and frequent as follows: alcohol 72.5%, tobacco 74.6%, ecstasy 41.3% and 25.4% cocaine. The use of cannabis was considered as a means of indulgence and pleasure for 66.7%, as an anxiolytic for 26.8% and as a sedative for 23.9%. Self-esteem, among those cannabis users, was very low in 20% of cases, low in 38% of cases, medium in 15% of cases and high in 25% of cases.

**Conclusions:**

These results lead us to question the relation between cannabis and self-esteem. The question that is evolved about the use of cannabis is the following: Is it used as a remedy or is it the cause of self-esteem deficiency?

**Disclosure:**

No significant relationships.

